# Prefrontal hyperactivation during dual-task walking related to apathy symptoms in older individuals

**DOI:** 10.1371/journal.pone.0266553

**Published:** 2022-04-25

**Authors:** Deborah Talamonti, Emma Gabrielle Dupuy, Sarah Boudaa, Thomas Vincent, Sarah Fraser, Anil Nigam, Frédéric Lesage, Sylvie Belleville, Christine Gagnon, Louis Bherer

**Affiliations:** 1 Research Centre and Centre EPIC, Montreal Heart Institute, Montreal, Canada; 2 Department of Medicine, Université de Montreal, Montreal, Canada; 3 Interdisciplinary School of Health Sciences, University of Ottawa, Ottawa, Canada; 4 École Polytechnique de Montréal, Montréal, Canada; 5 Centre de recherche, Institut universitaire de gériatrie de Montréal, Montréal, Canada; 6 Department of Psychology, Université de Montréal, Montréal, Canada; Preeminent Medical Phonics Education & Research Center, Hamamatsu University School of Medicine, JAPAN

## Abstract

Increasing evidence associates apathy with worsening in cognitive performance and greater risk of dementia, in both clinical and healthy older populations. In older adults with neurocognitive disorders, apathy has also been related to specific fronto-subcortical structural abnormalities, thus differentiating apathy and major depressive disorder. Yet, the neural mechanisms associated with apathy in healthy older adults are still unclear. In the present study, we investigated the frontal cortical response during a dual-task walking paradigm in forty-one healthy older adults with and without apathy symptoms, controlling for depressive symptoms. The dual-task walking paradigm included a single cognitive task (2-back), a single motor task (walking), and a dual-task condition (2-back whilst walking). The cortical response was measured by means of functional Near-Infrared Spectroscopy (fNIRS). The results revealed that participants with apathy symptoms showed greater activation of subregions of the prefrontal cortex and of the premotor cortex compared to healthy controls during the single cognitive component of the dual-task paradigm, whilst cognitive performance was equivalent between groups. Moreover, increased cortical response during the cognitive task was associated with higher odds of exhibiting apathy symptoms, independently of depressive symptoms. These findings suggest that apathy may be related to differential brain activation patterns in healthy older individuals and are in line with previous evidence of the distinctiveness between apathy and depression. Future research may explore the long-term effects of apathy on the cortical response in healthy older adults.

## Introduction

Apathy is defined as diminished motivation, reduced goal-directed behavior and emotional responsiveness [1] that occurs in a variety of neurological and psychiatric conditions [2], and in healthy individuals [2]. It constitutes a major public health problem [3], affecting 49% of healthy individuals over 77 years old [4], 60% of individuals with Alzheimer’s disease (AD), and 50% of those in pre-dementia stages [5]. Apathy has specific diagnostic criteria and neuroanatomic substrates that, although partially overlapping with major depressive disorders, clinically separate the two conditions [6–8]. Apathy in both healthy and clinical populations is associated with: decline in cognitive [2, 9] and physical functions [10], disability and frailty [10] and higher risk of dementia [11]. In clinical populations, apathy has also been related to altered brain structures. For instance, apathy is associated with alterations of the dopaminergic system in Parkinson disease; with Aβ depositions and reduced grey and white matter in AD, and to general cerebral atrophy in AD, stroke and HIV infection [12]. Across disorders, apathy has also been associated with structural changes in the dorsolateral prefrontal cortex and the basal ganglia [13]. These areas are involved in cognitive domains that are altered in apathy (e.g., goal-directed or motivated behaviors, emotional processing), thus suggesting that apathy may occur as a consequence of alterations in these brain systems [2]. Fewer attempts have been made to investigate its neuroanatomical mechanisms in not clinical populations, despite the importance of this topic for the formulation of ad-hoc and effective therapies. In a cohort study of over 4000 healthy older adults, an association between apathy symptoms and diffuse loss of both gray and white matter volumes, which was observed independent of depression, was found, thus corroborating previous findings from clinical populations [5]. Two studies investigated functional brain imaging phenotypes of apathy in healthy younger individuals. In these studies, apathy was linked to greater recruitment of premotor and prefrontal areas, as well as lower functional connectivity, during cognitive tests [14, 15]. The authors suggested that the greater activity may be due to inefficient prefrontal processing [15–17]. To the best of our knowledge, no study has to date explored brain activity related to apathy in healthy older individuals.

In the present study, we explored the frontal cortical response related to a test of dual-task walking in healthy older adults with and without apathy symptoms. We also investigated whether presence of apathy symptoms may be predicted based on cortical response in this population. The cortical response was recorded by means of a portable functional Near-Infrared Spectroscopy (fNIRS). fNIRS is an optical technique that monitors the task-related hemodynamic responses (concentration changes of oxygenated -HbO- and deoxygenated -HbR- hemoglobin) in the cortex [18]. Dual-task walking paradigms compare walking and cognitive tasks (e.g., counting back, naming animals, calculations) performed alone and simultaneously [19]. By recruiting brain resources shared by locomotor and cognitive control processes (i.e., prefrontal and temporal areas) [20], the dual-task walking limits the resources available for each task and may result in a decline in cognitive and/or walking performances [21]. Performance at the dual-task walking, as well as increased task-related cortical activity, have demonstrated their sensitivity to neurocognitive aging [19, 22]. Since the cortical areas related to both dual-task walking and apathy (i.e., motor and prefrontal regions) coincide, the dual-task walking may be especially suited to assess the task-related brain changes in individuals reporting apathy.

Given the existing evidence, in the present study it was hypothesized that those older adults reporting apathy symptoms would show greater prefrontal cortical response related to dual-task walking compared to asymptomatic controls. Specifically, since HbO signals tend to be more reliable overall than HbR signals [23], we expected greater HbO response in participants with apathy symptoms compared to controls. Moreover, we hypothesized that the HbO response would predict the probability of showing apathy symptoms.

## Materials and methods

The project was approved by the Montreal Heart Institute (MHI) ethic committee and completed in accordance with the Helsinki Declaration (protocol code: ICM#12–1386; date of approval: 07-18-2013) and all participants provided written consent to participate in the study. Participants were part of a longitudinal study investigating the relationship between regular physical activity, cardiovascular risk factors and cognitive decline and registered members of the preventive medicine and physical activity center (EPIC) of the MHI. Participants for this study were selected from the first visit if: aged 60 or above, were cognitively intact (Mini-Mental State Examination; MMSE > 26), and had no history of neurological, psychiatric or cardiovascular disease. These variables were either self-reported by participants during the clinical assessment (e.g., medical history), or collected during the neuropsychological assessment (e.g., MMSE) at the EPIC center. From the pool of sixty-eight participants, we analyzed baseline data from forty-one volunteers who fulfilled the above inclusion criteria. The 30-item Geriatric Depression Scale (GDS) [24] has been previously used in research to study apathy [8, 25, 26]. In the present study the GDS was used to assess both apathy and depressive symptoms. Inclusion in the apathy group was determined on the basis of having responded “yes” to at least one of the following items: (2) “Have you dropped many of your activities and interests?”; (12) “Do you prefer to stay at home rather than go out and do things?”; (20) “Is it hard for you to get started in new projects?”; (28) “Do you prefer to avoid social occasions?”. These items were selected following the definition of apathy form the latest edition of the diagnostic and statistical manual of mental disorders (DSM-V) [27] and through consensus between three co-authors (D.T., E.G.D., and C.G.), including a registered neuropsychologist (C.G.) and a researcher in neuropsychology (D.T.). Depressive symptoms were identified as the remaining 26-item on the GDS, which focused specifically on depressive symptomatology (e.g., (1)”Are you basically satisfied with your life?”; (15) “Do you think it is wonderful to be alive now?”; (25) “Do you frequently feel like crying?”).

The baseline visit consisted of a battery of tests investigating participants’ clinical, psychological and physical history and their cognitive abilities. The full longitudinal study has been described elsewhere [28]. Presence of cardiovascular risk factors (type 2 diabetes, hypertension, dyslipidemia, obesity, smoking, physical inactivity) was defined by a cardiologist during the clinical assessment and included both objective measures (e.g., blood pressure) and self-reported information (e.g., questions about smoking habits). Physical inactivity was defined in accordance with the World Health Organization 2020 guidelines on physical activity and sedentary behavior, that is, practising less than 150 minutes/week of moderate aerobic physical activity or 75 minutes/week of vigorous aerobic physical activity or an equivalent combination of the two [29].

Measures used for this study were as follows: MMSE [30], GDS [24], and the dual-task paradigm. The dual-task experimental design consisted of three conditions: single cognitive (SC), single-walking (SW), walking whilst completing the cognitive task (DT). The cognitive component of the dual-task consisted of a working memory task in which participants listened to series of numbers and then had to name the number presented two positions back (2-back). Participants heard one digit every 1.5 seconds through a Sennheiser headset. For the walking component of the dual-task participants were asked to freely walk on a 10 meters track back and forth for 30 seconds. The dual-task component consisted in completing the 2-back whilst walking. The dual-task experiment followed an ABBA design in order to control for fatigue effects, with trials presented in the following sequence: SC–SC–SW–DT–DT–DT–DT–SW–SC–SC. The experimental conditions were administered in blocks of 30 seconds, including a baseline period of 5 and 15 seconds, respectively before and after each block. For each cognitive trial, participants were presented 10 digits. If they were able to correctly respond to all 10-digit they received 100% accuracy score. Single cognitive (SC) trials were averaged for an overall accuracy percentage for each participant. Similarly dual-task (DT) trials were averaged to produce an overall DT accuracy percentage per participant. Gait speed was calculated by dividing the total distance walked (in meters) by the 30 seconds fixed time (in seconds). The m/s variable was calculated per each trial and then averaged over trials.

**Fig 1:**
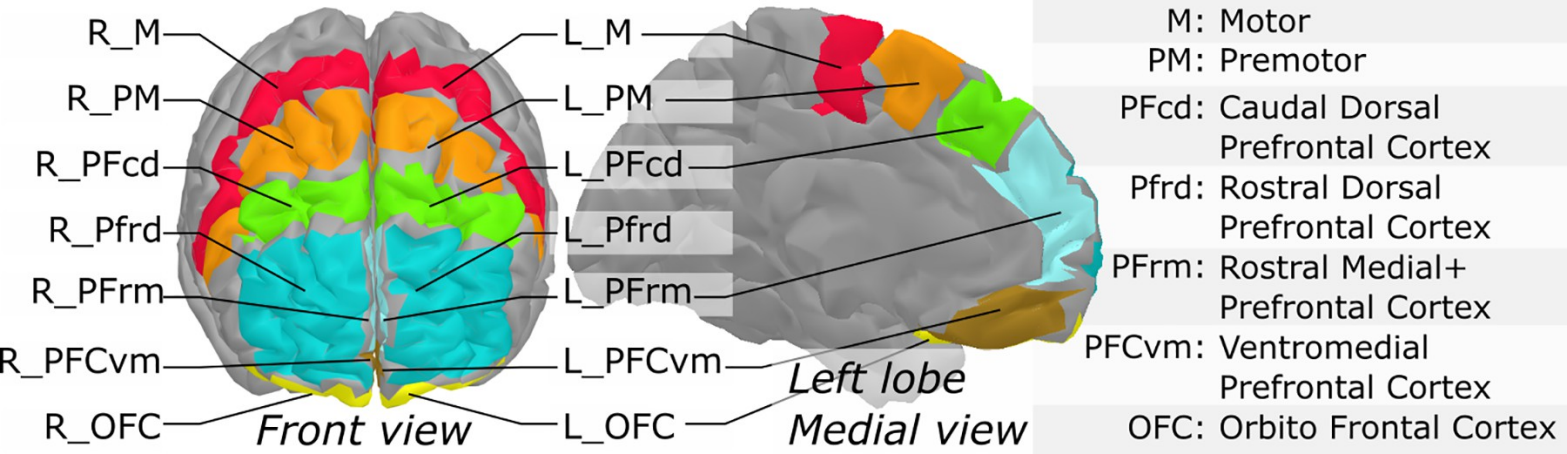
Segmentation of the prefrontal cortex based on MarsAtlas, used to produce region-averages of NIRS task-related effects.

During the dual-task walking, cortical response was recorded by means of a portable in-house built fNIRS system [31]. fNIRS light intensity signals (wavelengths 735nm and 860nm) were recorded at a sampling rate of 20 Hz. The fNIRS device consisted of a wireless portable system whose sensors covered the front of the head, with 16 sources and 16 detectors (256 channels). The optodes layout allowed to cover the prefrontal cortex (PFC) and part of the pre-motor areas. fNIRS signals were processed under Matlab using the brainstorm toolbox [32] and the nirstorm plugin [33]. The first step involved the removal of bad channels. Channels were considered bad when signals showed saturation periods, large changes or a high amount of noise without the presence of heart beats. Second, motion artefacts were manually tagged by identifying short-lived periods (max 2 sec) of abrupt changes spread across several channels. Motion correction was applied using a spline-based interpolation method [34], whereas a bandpass filtering between 0.01 and 0.1 Hz was applied to keep only the evoked hemodynamic band. Channel time-series were projected on the cortical surface of the Colin27 template [35] using the Minimum Norm Estimate algorithm [36]. The scalp-to-cortex projection matrix consisted of fluence values computed using an optical model [37] applied to a 5-tissue segmentation of the Colin27 MRI template (bone, blood, grey matter, skin and CSF). A first-level GLM with a pre-colored noise model [38] was applied to the cortical time-series of each subject to obtain within-subject t-stat mappings of [HbO] (oxygenated hemoglobin) and [HbR] (deoxygenated hemoglobin) task-evoked changes. Since the NIRS spatial resolution is relatively low, mesh-based cortical mappings contain redundant information. To get a more parsimonious representation of NIRS mappings, regional averages were computed using a coarse version of the MarsAtlas cortical parcellation [39]. This segmentation consisted of a set of 14 regions (7 per hemisphere) with the list of region labels (Fig 1). Lastly, to keep only the areas that were potentially engaged in the experimental paradigm, task-specific functional masks were computed from a group-level analysis. To do so, a second-level GLM with a mixed-effect noise model [38] was applied to produce binary maps from t-stats thresholded at p < .05 (uncorrected). For each experimental condition, this allowed to filter out the regions that elicited no activity at the group-level. Within-region and within-subject maps were computed as the effect size (estimated GLM effect magnitude divided by its standard deviation), to take into account temporal fluctuations that may vary across regions and participant.

For demographic and clinical variables, independent-samples t-tests and Fisher’s exact tests were run to explore between-group differences. For behavioral data (% accuracy and gait speed), intra and inter-individual differences were explored through two mixed 2 (group: apathy, control) × 2 (condition: single task, dual task) ANCOVAs, where sex and depressive symptoms were included as covariates, as the two groups differed on these variables. Following this, a series of one-way ANCOVAs were conducted to analyze group differences on each activated brain region (HbO and HbR responses) during each component of the dual-task paradigm (SC, SW, DT), whilst controlling for sex and depressive symptoms. Post-hoc pairwise comparisons were performed using Bonferroni correction. Finally, binary logistic regression models were performed to predict the probability of showing apathy symptoms based on the task-related hemodynamic response, during SC and DT respectively, with cortical responses as independent predictors in each regression and presence of apathy symptoms or not as the binary outcome. The regressions were adjusted for age, sex, education, and presence of depressive symptoms. Data were normally distributed, as assessed by Shapiro-Wilk test (p > .05). All other assumptions were met to perform the analyses.

## Results

Table 1 shows demographic, clinical characteristics and groups comparisons of participants. Data are mean ± standard deviation for continuous variables and count for categorial variable. Those classified as the control group were 24 (57.62%), whereas 17 (42.37%) had at least one apathy symptom. Of the total sample, 26 (63.40%) were females. Mean age was 66.93 ± 5.37 and mean level of years of schooling was 15.41 ± 3.38. All participants were cognitively healthy (MMSE = 28.41 ± 1.12). Participants with apathy symptoms were mostly females, *t*(39) = 2.190, *p* = .028 and reported greater depressive symptoms compared to the control group, *t*(39) = -3.327, *p* = .002. No other statistically significant group difference was found on demographic, clinical and behavioral variables (p-values reported in Tables 1 and S1).

**Table 1:** Demographic and clinical characteristics.

	Controls (N = 24)	Apathy (N = 17)	*p* value
**Female n (%)**	12 (50.0%)	14 (82.4%)	.028
**Age**	66.75 ± 5.45	67.18 ± 5.40	.806
**Education**	16.17 ± 2.68	14.35 ± 4.01	.090
**MMSE**	28.33 ± 1.17	28.53 ± 1.07	.586
**Depressive symptoms (26-item GDS)**	2.83 ± 2.96	6.18 ± 3.45	.002
**Cardiovascular risk factors:**			
**Diabetes n (%)**	3 (12.5%)	2 (11.8%)	.748
**Hypertension n (%)**	1 (4.2%)	1 (5.9%)	.360
**Dyslipidemia n (%)**	4 (16.7%)	3 (17.6%)	.818
**Obesity n (%)**	4 (16.7%)	4 (23.5%)	.596
**Smoking n (%)**	2 (8.3%)	5 (20.0%)	.219
**Physical inactivity n (%)**	2 (8.3%)	3 (17.6%)	.382

*Note*. Results are mean ± SD. MMSE = mini-mental state examination; GDS = geriatric depression scale.

The ANCOVA investigating group differences on the cognitive component of the dual-task (% accuracy) revealed no statistically significant main effects or interactions (*p* values > .05). The ANCOVA exploring group differences on the walking component of the dual-task (m/s) revealed no group difference, but a main effect of condition was found [F(1, 37) = 10.003, p = .003, η2p = .213], with walking speed being greater during SW (1.08 ± .149) than DT (1.02 ± .150), *p* < .001). There was no interaction between group and condition (*p* values > .05). Means and standard deviations of the dual-task paradigm are available in S1 Table.

**Fig 2:**
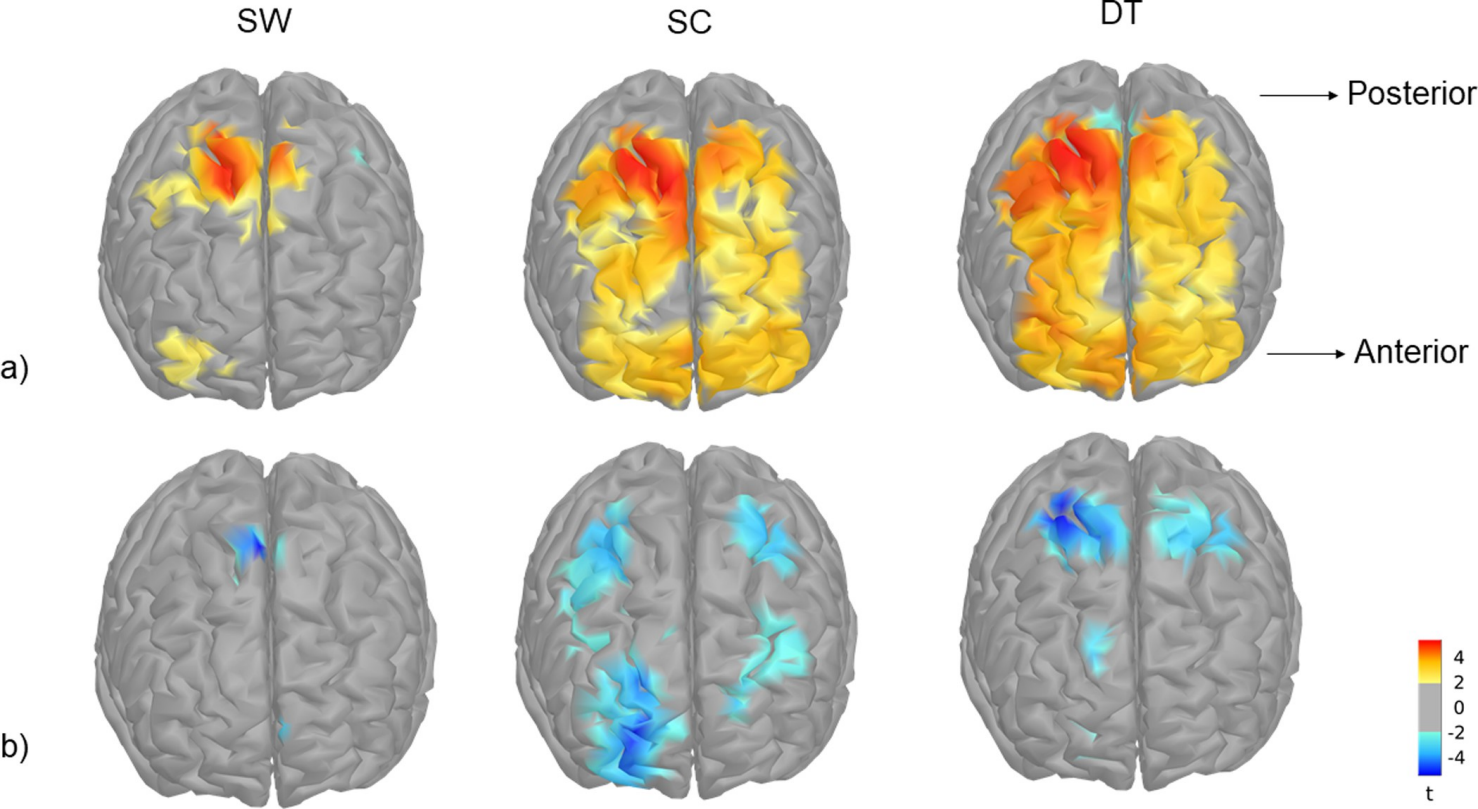
Second-level cortical mappings of HbO (a) and HbR (b) changes evoked by single walking (SW), single cognitive (SC) and dual-task (DT) conditions computed from all subjects and threhsolded at p < 0.05. Orientation: left is right.

The second-level GLM analysis, which provides a global functional mask of regions that are potentially involved in the task, showed bilateral activation of frontal areas. Cortical activation during SW was found mainly in the premotor and motor regions (PM, M) and in the caudal dorsal prefrontal region (PFcd) for HbO responses and in the premotor areas for HbR responses. During SC and DT, both prefrontal and motor regions were involved (PM, M, PFcd, rostral dorsal prefrontal -PFrd-, rostral medial prefrontal -PFrm-, and orbito-frontal cortex -OFC-) for HbO response, whereas HbR response was limited to PM, M, PFrd and PFcd (Fig 2).

**Fig 3:**
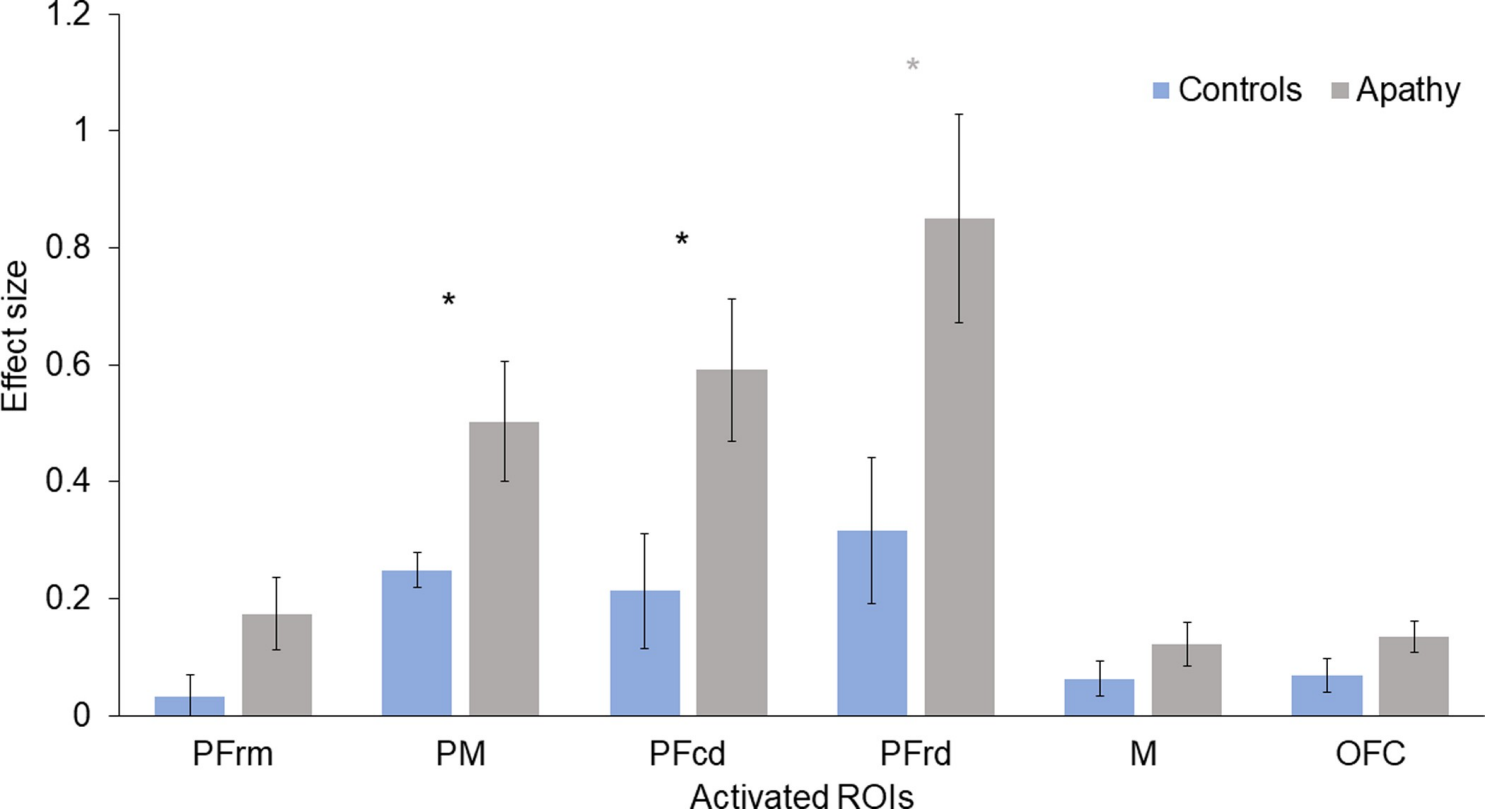
Effect sizes for HbO responses during the 2-back task (SC) in participants with or without apathy. Bars indicate standard error. PFrm = rostral medial prefrontal; PM = premotor; PFcd = caudal dorsal prefrontal; PFrd = rostral dorsal prefrontal; M = motor; OFC = orbito frontal cortex.

The ANCOVAs investigating group-related cortical activation (effect sizes) during SW and DT revealed no statistically significant results. The ANCOVAs exploring group differences on cortical response during the 2-back task (SC) revealed a main effect of group for the HbO responses in PM [F (1,37) = 5.679, p = .022, η2p = .133] and PFcd [F (1, 37) = 5.578, p = .024, η2p = .131], where the apathy group showed greater task-related cortical activation than the control group (Fig 3). No significant group differences were found for HbR responses. Means and standard errors of HbO and HbR responses are available in S2 Table.

Binomial logistic regressions were performed to investigate weather cortical response during SC and DT predicted apathy symptoms, whilst controlling for covariables. The models were statistically significant for the SC condition for PM [χ2(5) = 19.950, *p* = .001] and PFcd [χ2(5) = 19.796, *p* = .001] and explained 52% (Nagelkerke R^2^) of the variance in apathy symptoms, whilst correctly classifying 80% of cases in both models. Of the predictors, only two were statistically significant in the first model: presence of depressive symptoms and HbO response. In the second model the HbO response in PFcd tended to significance (p = .051) (Table 2). These results indicated that increased cortical response was associated with higher odds of exhibiting apathy symptoms. This association was significant even if depressive symptoms were also significantly associated with increased cortical response, as shown in Model 1.

**Table 2:** Logistic regression predicting apathy symptoms based on HbO responses in PM (Model 1) and PFcd (Model 2), adjusted for covariates.

	Model 1			Model 2		
	*B(SE)*	*p*	*OR*	*B(SE)*	*p*	*OR*
**Age**	.035 (.084)	.682	1.035	-.021 (.089)	.814	.979
**Sex**	-2.399 (1.288)	.063	.091	-2.159 (1.213)	.075	.115
**Years of education**	-.194 (.138)	.160	.823	-.200 (.142)	.157	.818
**Depressive symptoms**	.264 (.128)	.040	1.302	.233 (.126)	.066	1.262
**HbO response**	2.638 (1.337)	.049	13.980	2.101 (1.076)	.051	8.171

## Discussion

In this study, we investigated whether the presence of apathy symptoms in healthy older adults was related to increased cortical response during dual-task walking, comprising a single cognitive condition (2-back), a single walking condition, and a dual-task condition (2-back while walking). Compared to the control group, those reporting apathy symptoms showed greater cortical recruitment during the single cognitive condition (2-back), while both groups showed equivalent cognitive performance. This hyperactivation also predicted the presence of apathy symptoms.

fNIRS results showed activation of the prefrontal, premotor and motor areas. Premotor and motor areas were mainly activated during the motor component of the paradigm, whereas prefrontal and premotor regions were activated during the cognitive and the dual-task components of the paradigm. The aforementioned regions play a crucial role in both working memory [40] and walking [41], and their activation is in line with previous studies [42–44]. Interestingly, in the apathy group, we observed greater task-related cortical activation of the prefrontal dorsolateral and premotor regions during the single cognitive condition of the dual-task paradigm (2-back). The reduction of these areas in apathy is well-known in both healthy and clinical populations [4, 13, 15]. To date, no study had however compared task-related cortical response in healthy older adults with and without apathy symptoms. Previous comparisons in healthy younger individuals reported greater cortical activity of the dorsolateral prefrontal and premotor cortices during tasks of working memory and decision making, despite unaffected or worsened cognitive performance [14, 15]. Consistent with these findings, the larger cortical activation observed in the present study in older participants reporting apathy symptoms compared to those without, in the absence of any group difference in behavioral performance suggests an apathy-related reduction in neural resources that would cause a greater engagement (e.g., hyperactivation) of the prefrontal and premotor areas as a compensatory strategy aimed to avoid decline in cognitive performance. Such interpretation is in line with the compensation-related utilization of neural circuits hypothesis (CRUNCH), which suggests that increased cerebral activation acts as compensatory mechanism to avoid performance decline [45]. During the dual-task condition, group differences in cortical activity disappeared and greater cortical activation was reported in both groups indiscriminately. Given the increase in task difficulty in the dual-task condition, it may be that both controls and participants with apathy symptoms recruited more brain resources in order to sustain the greater task demands. This may have reduced the group differences observed during the condition with lower demand (SC). Although compensation mechanisms are considered functional in healthy older adulthood, as they may indicate brain plasticity [46], hyperactivation in populations at risk of AD (e.g., subjective cognitive decline) has been suggested as an early marker of AD, which may precede hypoactivation and measurable cognitive deficits [47, 48]. Similarly, greater frontal activation related to working memory tasks was observed in another population at risk of AD (e.g., mild cognitive impairment) [49]. It may thus be that the hyperactivation observed in our apathy group may as well reflect compensatory mechanisms and potentially be an early biomarker of cognitive impairment and dementia. Future research may consider shedding light on the functional brain changes that precede or predict the cognitive decline documented in this population in order to confirm such hypothesis. Our findings also contribute to further separate apathy from depression, given that under- rather than over-activation of the frontal areas is typically observed in clinical depression [50, 51] and in healthy individuals with depressive symptomatology [52].

On a behavioral level, no group differences were detected in any condition of the dual-task. Although lower cognitive and physical performances were reported in previous investigations [2, 10], it has to be noted that participants in those studies presented greater or more severe apathy symptomatology than our sample [2, 10] and that in the present study, the size of the sample was significantly smaller, thus potentially reducing our statistical power. Moreover, although lower cognitive performance, including in the domain of working memory, has been observed repeatedly in clinical populations with apathy [9, 25, 53], studies in healthy older populations are to date very limited. Our findings therefore suggest that there may be a limit in the use of cognitive measures to detect the effects of apathy in healthy older individuals, although further research is needed to better understand the impact of apathy in non-clinical populations.

Some limitations to this study should be taken into account. Firstly, our results are based on self-reported apathy symptomatology, rather than clinical apathy and standardized apathy scales, and do not include information regarding history of diagnosed mood disorders. Secondly, results from a cross-sectional study do not allow to conclude on the causal link between apathy and brain activation. Thus, future research may explore the longitudinal impact of apathy on the brain in relation to cognitive decline. Moreover, future studies may consider the use of a comprehensive battery of neuropsychological tests to better assess specific cognitive domains. Third, the findings from HbO responses did not match those from HbR responses. It has to be noted, however, that the HbR responses were less pronounced than HbO responses in our study (in line with previous studies; [54], which typically cause greater changes in amplitude. HbO responses are therefore believed to better reflect neurovascular coupling [18]. This point introduces our final limitation, which was the use of fNIRS to investigate the neural correlates of apathy. Although fNIRS is a remarkable technique in walking research and has several advantages for both cognitive and clinical research, its limited and superficial coverage of the brain necessitates that our results are confirmed through the use of techniques that do not pose these limitations (e.g., fMRI). Moreover, future investigations may consider replicating our results by recruiting an equivalent proportion of male and female participants, in order to better understand the influence of biological sex on brain patterns in apathy.

The findings of this study demonstrated less efficient prefrontal processing during a task of working memory in healthy older adults with apathy symptoms compared to controls, and shed new light on the relationship between apathy and cortical activity. These results may be crucial for tailored approaches that prevent clinical apathy in the older population and, consequently, cognitive decline. For instance, it should be taken into consideration that apathy symptoms may not necessarily affect the performance at cognitive tests during routine visits, but still impact brain health in the long term. Alternatively, given the extensive number of investigations using brain stimulation techniques to treat mood disorders [55], the regions investigated in the present and previous studies may be selected in trials aiming at preventing clinical apathy by means of brain stimulation technologies.

## Supporting information

S1 Table Means and standard deviations of behavioral data during each experimental condition. (DOCX)

S2 Table Means and standard errors of effect sizes for HbO and HbR responses evoked by the 2-back task (SC). (DOCX)
