# Supplementary material for: Prefrontal hyperactivation during dual-task walking related to apathy symptoms in older individuals
Source: PLoS One. 2022 Apr 25;17(4):e0266553. doi: 10.1371/journal.pone.0266553 (PMC9037904; doi:10.1371/journal.pone.0266553)
Supplement: S1 Table — (DOCX) [file pone.0266553.s001.docx]

**S1 Table.**

|  | **Controls (N = 24)** | **Apathy (N = 17)** | ***p* value** |
| --- | --- | --- | --- |
| **Cognitive performance:** | |  |  |
| **SC** | 76.25 ± 17.38 | 74.56 ± 23.49 | .792 |
| **DT** | 77.71 ± 19.77 | 80.73 ± 20.34 | .803 |
| **Gait Speed:** |  |  |  |
| **SW** | 1.09 ± .167 | 1.08 ± .118 | .844 |
| **DT** | 1.02 ± .168 | 1.03 ± .132 | .823 |

*Note.* SC = single cognitive condition; SW = single walking condition; DT = dual-task condition.
