# Supplementary material for: Prefrontal hyperactivation during dual-task walking related to apathy symptoms in older individuals
Source: PLoS One. 2022 Apr 25;17(4):e0266553. doi: 10.1371/journal.pone.0266553 (PMC9037904; doi:10.1371/journal.pone.0266553)
Supplement: S2 Table — (DOCX) [file pone.0266553.s002.docx]

**S2 Table**.

| **ROIs** | **HbO response** | | **HbR response** | |
| --- | --- | --- | --- | --- |
|  | *Controls* | *Apathy group* | *Controls* | *Apathy group* |
| PM | **.249 ± .029** | **.503 ± .103** | -.257 ± .053 | -.173 ± .051 |
| M | .063 ± .030 | .122 ± .037 | -.061 ± .017 | -.067 ± .024 |
| PFrm | .032 ± .038 | .174 ± .062 | - | - |
| PFrd | .317 ± .125 | .850 ± .178 | -.393 ± .114 | -.249 ± .111 |
| PFcd | **.213 ± .099** | **.591 ± .121** | -.257 ± .053 | -.111 ± .073 |
| OFC | .069 ± .029 | .135 ± .026 | - | - |

*Note.* Values shown in Δumol/L. Highlighted difference between groups p<0.05. HbR responses in PFrm and OFC were not observed.
